# Radiological Findings in Reversible Cerebral Vasoconstriction Syndrome: A Systematic Review of Literature

**DOI:** 10.7759/cureus.59595

**Published:** 2024-05-03

**Authors:** Gayathri M Sivagurunathan, Anas Khan, Dimitrios Fotopoulos

**Affiliations:** 1 Radiology, Indira Gandhi Medical College & Research Institute, Pondicherry, IND; 2 Radiology, University Hospitals of Morecambe Bay NHS Foundation Trust, Lancaster, GBR; 3 Internal Medicine, University Hospitals of Morecambe Bay NHS Foundation Trust, Lancaster, GBR

**Keywords:** ct, mri, blood-brain barrier disruption, cerebral dysautoregulation, vascular wall enhancement, systematic review, radiology, reversible cerebral vasoconstriction syndrome

## Abstract

Reversible cerebral vasoconstriction syndrome (RCVS) poses a complex neurological challenge characterized by sudden, severe headaches and multifocal cerebral vasoconstriction. While our understanding of its clinical aspects and underlying mechanisms has advanced, the focus of investigation remains on radiological manifestations. This systematic review aims to comprehensively analyze the existing literature on radiological findings in RCVS, synthesizing evidence from diverse imaging modalities to enhance the understanding of imaging features associated with the syndrome. Accurate diagnosis based on radiological findings is pivotal for initiating appropriate management and preventing complications. Specific markers may facilitate the differentiation of RCVS from other conditions, thereby enhancing patient care. This review explores a wide range of radiological presentations, from vasoconstriction to infarctions and hemorrhages, thereby refining diagnostic criteria and guiding clinical practice. By consolidating current knowledge, the review sheds light on areas of consensus, controversies, and gaps, with the aim of serving as a comprehensive resource for evidence-based decision-making.

## Introduction and background

Reversible cerebral vasoconstriction syndrome (RCVS) is an intriguing yet challenging neurological disorder characterized by thunderclap headaches and reversible multifocal cerebral vasoconstriction, often associated with neurological deficits and complications such as ischemic or hemorrhagic stroke [1]. While the clinical presentation and pathophysiology of RCVS have been increasingly elucidated, the radiological manifestations remain a subject of intense investigation. The utilization of various imaging modalities has provided invaluable insights into the pathogenesis, diagnosis, and management of RCVS [2]. This systematic review aims to analyze the existing literature on radiological findings in RCVS, drawing evidence from various imaging techniques such as magnetic resonance imaging, magnetic resonance angiography (MRA), computed tomography angiography (CTA), transcranial Doppler, and others. The goal is to improve our understanding of the imaging features associated with the syndrome.

Furthermore, elucidating the radiological patterns and abnormalities encountered in RCVS holds immense clinical significance. Accurate and timely diagnosis based on radiological findings is pivotal for initiating appropriate management strategies, prognostication, and preventing potentially devastating complications [3,4]. In this systematic review, we will explore the diversity of radiological presentations observed in RCVS patients, ranging from segmental vasoconstriction to cerebral infarctions, intracerebral hemorrhage, and various associated complications. By critically evaluating the existing literature, we aim to elucidate the prevalence, characteristics, and prognostic implications of different radiological findings in RCVS, thereby contributing to the refinement of diagnostic criteria and informing clinical practice.

Overall, this systematic review aims to consolidate the current knowledge base on radiological manifestations in RCVS, highlighting areas of consensus, controversies, and gaps in understanding. By synthesizing evidence from a multitude of studies, we aspire to provide clinicians and researchers with a comprehensive resource that facilitates evidence-based decision-making, guides future research endeavors, and ultimately improves patient outcomes in this enigmatic neurological disorder.

## Review

Methods

Study Design and Search Strategy

The protocol for this systematic review has been registered on a publicly accessible platform, PROSPERO (CRD42024521697), outlining the planned methodology and analysis. A comprehensive search was conducted from the last decade (2014 -2024) using the PubMed database, to identify relevant studies. The search terms “Reversible cerebral vasoconstriction syndrome AND radiological imaging” were used to gather relevant literature.

Inclusion Criteria

Studies were deemed eligible for inclusion if they met the following predefined criteria: publication in peer-reviewed journals; reporting of radiological findings in patients diagnosed with RCVS; utilization of any imaging modality, including, but not limited to, magnetic resonance angiography (MRA), computed tomography angiography (CTA), digital subtraction angiography (DSA), magnetic resonance imaging (MRI), transcranial Doppler (TCD), and computed tomography (CT); provision of clear descriptions of radiological findings related to RCVS, encompassing segmental vasoconstriction, cerebral infarction, intracerebral hemorrhage, cortical subarachnoid hemorrhage, and other associated complications; focus on adult populations; and publication in any language, with availability of English translation.

Exclusion Criteria

The studies were excluded if they lacked clear reporting of radiological findings or primarily focused on clinical aspects without providing detailed radiological characterization. Additionally, case reports, case series, letters, editorials, and review articles lacking original data were precluded from consideration. To maintain specificity to RCVS, studies not directly pertinent to RCVS or solely addressing other neurovascular disorders were disregarded. Furthermore, studies with a sample size of fewer than five participants were omitted from the analysis. Studies lacking full-text availability or where relevant data were inaccessible were systematically excluded. To avoid redundancy, duplicate publications or redundant data from the same study population were not considered, with preference given to the most comprehensive or recent study. Moreover, studies conducted on animal models or in vitro settings were not considered to maintain clinical relevance. Additionally, studies solely focusing on treatment outcomes or prognostic factors without substantial radiological assessment were disregarded to uphold the study's diagnostic specificity. Lastly, studies with significant methodological flaws or a high risk of bias, as determined by the quality assessment process, were also excluded from the analysis.

Study Selection

**Figure 1:**
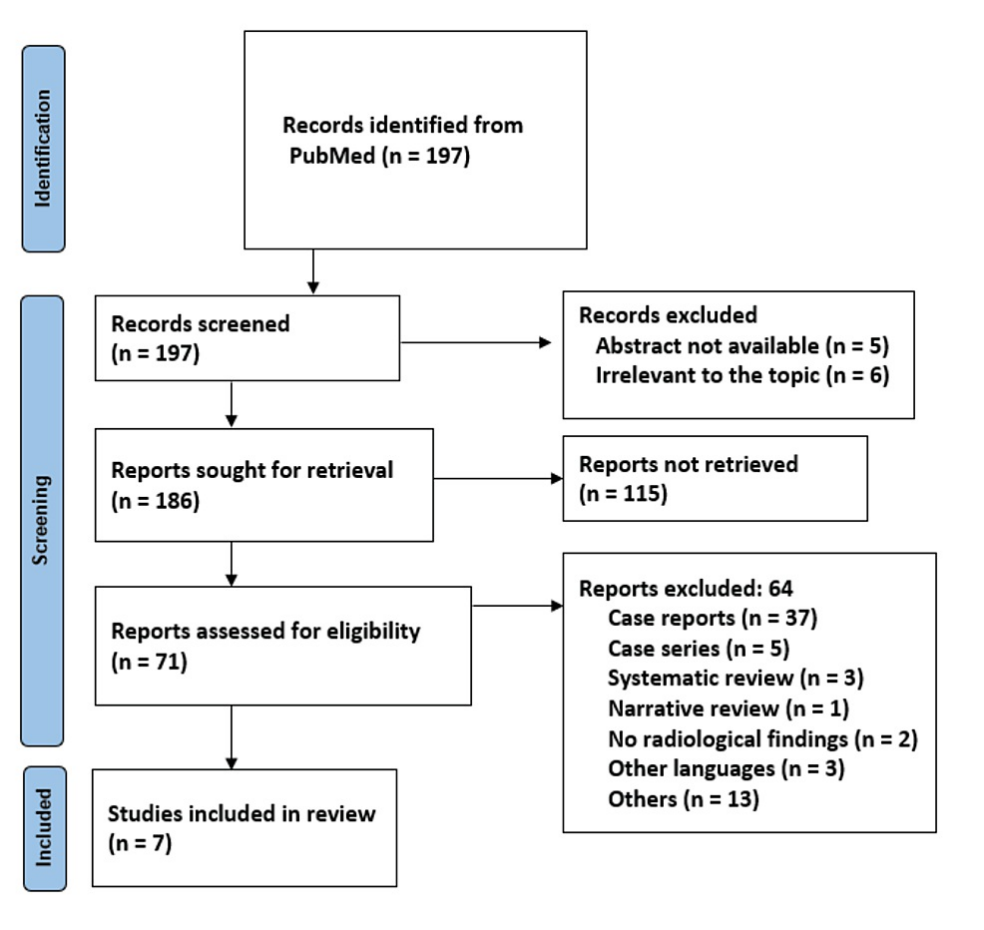
PRISMA flowchart illustrating the study selection process. PRISMA: Preferred Reporting Items of Systematic Reviews and Meta-Analysis

Two independent reviewers initially screened the titles and abstracts of all identified studies against the predefined inclusion and exclusion criteria. Studies deemed eligible had their full texts retrieved for further assessment. The same two reviewers independently assessed the full texts of potentially eligible studies to determine final inclusion. Any discrepancies or disagreements between reviewers were resolved through discussion or consultation with a third reviewer. A flow diagram was generated to illustrate the study selection process, adhering to Preferred Reporting Items of Systematic Reviews and Meta-Analysis (PRISMA) guidelines (Figure 1). Reasons for excluding studies at each stage of the selection process were documented.

Search Results

An initial search of the PubMed database yielded 197 documents. Following screening, 11 articles were excluded, leaving a total of 186 articles for full-text analysis. However, among these, 115 were discarded due to the unavailability of free text. Subsequently, the eligibility of the remaining 71 articles was assessed, resulting in the exclusion of 64 articles. Among the excluded articles, 37 were case reports, five articles were case series, three articles were systematic reviews, one was a narrative review, and two lacked radiological findings. Additionally, three articles were in languages other than English, and 13 articles did not meet the eligibility criteria. Consequently, seven articles were deemed suitable and included in the review (Figure 1).

Data Extraction

Data extraction involved capturing various data items, including study characteristics such as authors, publication year, country of origin, study design, and sample size. Information regarding the imaging modalities used and radiological findings was also gathered. The extraction process was conducted by two independent reviewers utilizing a standardized form, with any discrepancies resolved through discussion or consultation with a third reviewer.

Quality Assessment

Quality assessment was performed, with the risk of bias assessed using the Newcastle-Ottawa Scale for observational studies.

Data Synthesis

Data synthesis involved a narrative synthesis of findings, which summarized the characteristics and prevalence of radiological findings in RCVS across the included studies. The overall strength of evidence was evaluated using GRADE (Grading of Recommendations Assessment, Development, and Evaluation) criteria.

Results

The results of the study are as follows: Table 1 shows the overview of all the included studies. The table encompasses a range of study designs, including case-control studies, retrospective studies, and prospective studies. This diversity in study designs reflects the varied methodologies employed to investigate radiological findings in RCVS. The publication years span from 2014 to 2024, indicating a relatively recent body of research on this topic. The inclusion of studies published over the past decade ensures a comprehensive synthesis of the latest evidence in the field. The sample sizes of included studies vary widely, ranging from smaller-scale studies with sample sizes as low as 10 participants to larger-scale investigations with sample sizes exceeding 100 individuals. Geographic locations of the studies include Taiwan, the USA, South Korea, and Germany, highlighting the global interest and research efforts dedicated to understanding radiological manifestations in RCVS across different regions.

**Table 1:** Overview of the included studies. BBB: Blood-brain barrier.

Study	Title of the study	Publication year	Study design	Sample size	Geographic location
Ling et al. [5]	Association between impaired dynamic cerebral autoregulation and BBB disruption in reversible cerebral vasoconstriction syndrome	2023	Cross-sectional study	Cases – 45; Controls – 45	Taiwan
Singhal et al. [6]	Glucocorticoid-associated worsening in reversible cerebral vasoconstriction syndrome	2017	Retrospective study	162	USA
Choi et al. [7]	Cerebral endothelial dysfunction in reversible cerebral vasoconstriction syndrome: a case-control study	2017	Case-control study	8: RCVS – 28; Migraine-28; Controls – 28	South Korea
Wu et al. [8]	Dynamic changes in glymphatic function in reversible cerebral vasoconstriction syndrome	2024	Prospective study	Cases – 138; Controls – 42	Taiwan
Cheng et al. [9]	A common cause of sudden and thunderclap headaches: reversible cerebral vasoconstriction syndrome	2014	Prospective study	31	Taiwan
Kraayvanger et al. [10]	Cerebrospinal fluid findings in reversible cerebral vasoconstriction syndrome: a way to differentiate from cerebral vasculitis?	2018	Retrospective study	10	Germany
Chen et al. [11]	Vascular wall imaging in reversible cerebral vasoconstriction syndrome – a 3-T contrast-enhanced MRI study	2018	Prospective study	62	Taiwan

Table 2 lists various radiological imaging modalities, including CT, MRI, vessel wall MRI imaging, DSA, CTA, MRA, magnetic resonance venography (MRV), TCD, and diffusion tensor imaging (DTI). Each cell in the table indicates whether the corresponding imaging modality was employed in the respective study (denoted by "X") or not. The last row of the table presents a tally of the total number of studies utilizing each imaging modality. For example, TCD was employed in five out of the total number of studies, MRI in all seven studies, DSA in one study, and so on. This provides insights into the prevalence and utilization of different imaging techniques in the investigation of radiological findings in reversible cerebral vasoconstriction syndrome. The data indicate variability in the selection and utilization of imaging modalities across the included studies. Some studies utilized multiple imaging modalities, while others focused on specific techniques. For example, MRI was the most commonly employed imaging modality, utilized in all the studies. The diversity of imaging modalities employed reflects a comprehensive approach to radiological assessment in RCVS, aiming to capture various aspects of cerebral vasculature, perfusion, and parenchymal abnormalities. This comprehensive approach enhances the ability to characterize and understand the radiological manifestations of RCVS across different imaging techniques and modalities. 

**Table 2:** Radiological imaging modalities. "X" denotes the corresponding imaging modality was employed in the respective study. CT: Computed tomography; MRI: Magnetic resonance imaging; DSA: Digital subtraction angiography; CTA: Computed tomography angiography; MRA: Magnetic resonance angiography; MRV: Magnetic resonance venography; DTI: Diffusion tensor imaging

Study	CT	MRI	DSA	CTA	MRA	MRV	Transcranial Doppler	DTI
Ling et al. [5]		X		X	X		X	
Singhal et al. [6]		X	X	X	X			
Choi et al. [7]		X			X		X	
Wu et al. [8]		X			X		X	X
Cheng et al. [9]	X	X (in addition vascular wall MRI imaging also performed)		X	X			
Kraayvanger et al. [10]		X		X	X		X	
Chen et al. [11]		X			X	X	X	
Total	1	7	1	4	7	1	5	1

Table 3 presents the prevalence of various radiological findings observed in the included participants across the studies in the systematic review. The table lists several radiological findings commonly associated with reversible cerebral vasoconstriction syndrome, including infarct, parenchymal hemorrhage, convexal subarachnoid hemorrhage (SAH), and posterior reversible encephalopathy syndrome (PRES). Infarct emerges as the most prevalent radiological finding among the included participants, with prevalence rates of 35%. The prevalence rates of the radiological findings provide insights into the frequency and distribution of cerebral vascular and parenchymal abnormalities associated with RCVS. According to Singhal et al. [6], there was a correlation between clinical deterioration and worsening angiographic findings. Additionally, the subset of patients who experienced clinical deterioration exhibited a higher prevalence of involvement of intracranial arteries [6] (refer to Table 4).

**Table 3:** Prevalence of radiological findings. SAH: Subarachnoid hemorrhage; PRES: Posterior reversible encephalopathy syndrome

Radiological findings	Total number of participants n=231 (%) (Based on the studies by Singhal et al. [6], Choi et al. [7], Cheng et al. [9], and Kraayvanger et al. [10])
Infarct	80 (35%)
Parenchymal hemorrhage	36 (15%)
Convexal SAH	26 (11%)
PRES	38 (16%)

**Table 4:** Distribution of intracranial arteries involvement in RCVS. RCVS: Reversible cerebral vasoconstriction syndrome; ICA: internal carotid artery

MR angiogram findings (vasoconstriction)	Total number of participants n=162 (%) (Based on the study by Singhal et al. [6])	
	Clinically worsened (n=23)	Not worsened (n=139)
Intracranial ICA involved	35%	13%
Middle cerebral artery involved	87%	89%
Anterior cerebral artery involved	100%	82%
Posterior cerebral artery involved	91%	77%
Vertebral or basilar artery involved	70%	45%

It's important to interpret these prevalence rates within the context of the included studies and their methodologies. Variations in imaging techniques, patient populations, and study designs may influence the observed prevalence rates, and caution should be exercised when generalizing findings to broader populations.

Table 5 presents the results of the quality assessment and risk of bias for each included study using the Newcastle-Ottawa Scale. The table outlines the scores for selection, comparability, and outcome domains, with maximum scores of 4, 2, and 3, respectively.

**Table 5:** Quality assessment and risk of bias.

Study	Selection (out of 4)	Comparability (out of 2)	Outcome (out of 3)
Ling et al. [5]	3	2	3
Singhal et al. [6]	4	2	3
Choi et al. [7]	2	1	2
Wu et al. [8]	3	2	3
Cheng et al. [9]	4	2	3
Kraayvanger et al. [10]	2	1	2
Chen et al. [11]	4	2	3

Selection Domain

Studies by Ling et al. [5], Wu et al. [8], Cheng et al. [9], and Chen et al. [11] received scores of 3 or 4, indicating moderate to high quality in participant selection methods. Additionally, the study by Singhal et al. [6] also received a high score of 4, suggesting robust participant selection criteria. However, studies by Choi et al. [7] and Kraayvanger et al. [10] received lower scores of 2, indicating potential limitations in participant selection methods.

Comparability Domain

Studies by Singhal et al. [6], Wu et al. [8], Cheng et al. [9], and Chen et al. [11] received scores of 2, signifying their adequate consideration of group comparability or adjustment for confounding factors. However, studies by Choi et al. [7] and Kraayvanger et al. [10] received scores of 1, indicating limited comparability or adjustment for confounders. The study by Ling et al. [5] received a score of 2, suggesting moderate comparability between groups.

Outcome Domain

Studies by Choi et al. [7] and Kraayvanger et al. [10] received scores of 2 and the remaining studies received scores of 3 in the outcome domain, suggesting robust assessment methods and minimal risk of bias in outcome measurement. This indicates that the studies adequately measured outcomes of interest and minimized bias in outcome assessment.

Discussion

The systematic review encompasses a wide range of study designs, reflecting the comprehensive approach to investigating radiological findings in RCVS. Case-control, retrospective, and prospective studies have all enriched our understanding, providing distinct insights into the radiological aspects of RCVS. The inclusion of studies spanning from 2014 to 2024 underscores the recent surge in interest and advancements in this domain. Additionally, the global distribution of studies, including contributions from Taiwan, the USA, South Korea, and Germany, underscores the universal importance of RCVS and the collaborative endeavors of researchers worldwide.

Table 2 provides a comprehensive overview of the radiological imaging modalities employed across the included studies. MRI emerges as the most frequently utilized modality. This emphasis on MRI underscores its utility in capturing various aspects of cerebral vasculature and parenchymal abnormalities associated with RCVS. The variability in imaging modalities reflects the complex nature of RCVS and the need for a multi-modality approach for comprehensive radiological assessment.

Table 3 outlines the prevalence of radiological findings observed in RCVS patients across the included studies. Infarct emerges as the most prevalent finding. Subarachnoid and parenchymal hemorrhages and posterior PRES are also observed. These findings underscore the heterogeneity of radiological presentations in RCVS and the importance of thorough imaging evaluation in clinical practice.

Table 5 provides a critical appraisal of the methodological quality and risk of bias in the included studies using the Newcastle-Ottawa Scale. Overall, the majority of studies demonstrate moderate to high quality in participant selection and outcome assessment. However, there are variations in the comparability of groups and adjustments for confounding factors across studies. This highlights the need for careful consideration of potential biases when interpreting the results and underscores the importance of robust study design in advancing our understanding of RCVS.

The findings of the systematic review have important implications for clinical practice. Understanding the radiological manifestations of RCVS is crucial for accurate diagnosis, prognostication, and treatment planning. Radiological features such as infarction and SAH can guide clinicians in distinguishing RCVS from other cerebral vascular disorders and tailoring management strategies accordingly [12]. Furthermore, the identification of specific imaging markers associated with disease severity or prognosis may aid in risk stratification and therapeutic decision-making. Beyond their clinical relevance, the radiological findings elucidated in this review provide valuable insights into the underlying pathophysiology of RCVS (Table 6).For instance, in the acute stage of RCVS, the glymphatic function is lower but increases during recovery from vasoconstriction. The DTI-ALPS index, which measures glymphatic function, is lower when headaches are severe, suggesting a link between glymphatic dysfunction and symptom severity [8]. Similarly, the correlation between dynamic cerebral autoregulation and blood-brain barrier disruption suggests intricate interactions between cerebrovascular dynamics and vascular permeability in RCVS [13]. PRES can occur concurrently with RCVS [14], and while the precise etiologies of both conditions remain topics of debate, they share common risk factors and symptoms. This implies a potential shared origin related to cerebral autoregulation, endothelial dysfunction [15,16], and compromise of the blood-brain barrier [17-20]. Such insights contribute to our evolving comprehension of RCVS and may inform about the development of improved treatment strategies.

**Table 6:** Radiological patterns and characteristics. RCVS: Reversible cerebral vasoconstriction syndrome; RI: Resistive index; dCA: Dynamic cerebral autoregulation; DTI-ALPS: Diffusion tensor image analysis along the perivascular space

Study	Radiological characteristics
Chen et al. [11]	Vascular Wall Enhancement Characteristics: Vascular wall imaging during the ictal stage revealed enhancement in 45.8% of patients, with 22.7% showing marked enhancement and 77.3% showing mild enhancement. The enhancement was observed to be both concentric (72.7%) and eccentric (27.3%), with the eccentric pattern mainly associated with mild enhancement. Segmental location analysis indicated involvement of proximal M1 of the middle cerebral artery in 13.6%, distal M1 in 27.3%, and the whole M1 segment in 59.1% of cases. There was no significant difference in maximal flow velocity between patients with and without vascular wall enhancement. Half of the RCVS patients exhibited enhancement of diseased vessels, with one-third of them showing persistent enhancement. Consequently, vascular wall enhancement may not serve as a reliable imaging marker for distinguishing RCVS from central nervous system vasculitis or subclinical atherosclerosis. [11]
Choi et al. [7]	Transcranial Doppler (TCD) Findings in RCVS: Patients with RCVS demonstrated higher baseline mean flow velocities (MFVs) in bilateral middle cerebral arteries (MCA) and the basilar artery (BA) compared to healthy controls and individuals with episodic migraine without aura (MOA). Some RCVS patients exhibited increased MFVs exceeding the normal range, with a subset meeting the criteria for mild vasospasm. Additionally, RCVS patients showed lower breath-holding indices (BHIs) in basal arteries compared to healthy controls, indicating impaired cerebral endothelial function in RCVS.
Ling et al. [5]	Association between blood-brain barrier (BBB) disruption and cerebral dysautoregulation: RCVS patients with image-proven BBB disruption showed the worst dynamic cerebral autoregulation (dCA) compared to those without BBB disruption and healthy controls. Specifically, RCVS patients with BBB disruption exhibited the smallest very-low frequency (VLF) phase shift and largest mean flow correlation index (Mx), indicative of more severe dysautoregulation. Older age, history of migraine, and specific dCA metrics were associated with BBB disruption, highlighting potential clinical predictors.
Wu et al. [8]	Association between distal internal cerebral arteries RI and DTI-ALPS Index: The vascular investigations demonstrated a negative correlation between the resistance index (RI) of the distal internal carotid artery (ICA) and the DTI-ALPS index in all RCVS subjects, indicating heightened distal resistance due to intracranial vasoconstriction. This vasoconstriction, coupled with dysregulated autonomic activity, likely reduces pulsatility in distal vessels, potentially impeding glymphatic flow. The positive correlation between mean middle cerebral artery (MCA) flow and the DTI-ALPS index post-onset suggests a disruption in glymphatic flow and vasomotor control coupling during the acute stage, which may restore with vasoconstriction and autonomic dysfunction improvement in later stages of RCVS.

While the systematic review consolidates existing evidence, several challenges and avenues for future research warrant consideration. Methodological heterogeneity among studies, including variations in imaging protocols and diagnostic criteria, lack of clinical suspicion, and lack of follow-up imaging studies, poses challenges to data synthesis and interpretation.

Standardization of imaging methodologies and consensus guidelines for RCVS diagnosis and classification are essential to address these challenges. Additionally, prospective longitudinal studies with larger cohorts are needed to validate imaging findings, elucidate disease trajectories, and identify biomarkers predictive of clinical outcomes. Given the multifaceted nature of RCVS, multidisciplinary collaboration between neurologists, radiologists, vascular specialists, and other healthcare professionals is imperative for accurate diagnosis. Furthermore, collaborative research initiatives and data-sharing platforms enable the aggregation of diverse datasets, fostering a collective understanding of RCVS and accelerating scientific progress.

## Conclusions

This systematic review comprehensively examines the radiological findings in RCVS, synthesizing evidence from a diverse array of studies spanning different geographic locations, study designs, and imaging modalities. Through meticulous analysis, several key observations emerge, shedding light on the clinical, pathophysiological, and diagnostic aspects of RCVS. The overview of included studies reveals a burgeoning interest in RCVS research over the past decade, reflected in a breadth of investigations employing varied methodologies. From case-control studies to prospective cohorts, researchers have endeavored to unravel the intricate radiological manifestations of RCVS, contributing to a nuanced understanding of the disease. Radiological imaging modalities play a pivotal role in the diagnosis and characterization of RCVS, with MRI emerging as a cornerstone modality alongside CT, angiography, and Doppler techniques. These modalities offer invaluable insights into cerebrovascular dynamics, perfusion abnormalities, and parenchymal changes associated with RCVS, enabling clinicians to make timely and accurate diagnoses. Prevalence data highlight the spectrum of radiological findings encountered in RCVS from infarcts to PRES. These findings underscore the heterogeneity of RCVS presentations and the importance of comprehensive imaging evaluations in clinical practice. This systematic review advances our knowledge of radiological findings in RCVS, providing clinicians and researchers with a comprehensive framework for understanding and managing this complex cerebrovascular disorder. By integrating evidence-based insights into clinical practice and fostering ongoing research endeavors, we can enhance diagnostic accuracy, refine prognostic models, and ultimately improve outcomes for individuals affected by RCVS.
